# Low adherence to a new guideline for managing febrile infants ≤59 days

**DOI:** 10.3389/fped.2024.1401654

**Published:** 2024-06-04

**Authors:** Matilda Elliver, Josefin Norrman, Ioannis Orfanos

**Affiliations:** ^1^ Department of Clinical Sciences, Lund University, Lund, Sweden; ^2^ Department of Pediatrics, Skåne University Hospital, Lund, Sweden

**Keywords:** adherence, febrile infants, guideline, lumbar puncture, serious bacterial infection

## Abstract

**Background:**

Management of young febrile infants is challenging. Therefore, several guidelines have been developed over the last decades. However, knowledge regarding the impact of introducing guidelines for febrile infants is limited. We assessed the impact of and adherence to a novel guideline for managing febrile infants aged ≤59 days.

**Methods:**

This retrospective cross-sectional study was conducted in 2 pediatric emergency departments in Sweden between 2014 and 2021. We compared the management of infants aged ≤59 days with fever without a source (FWS) and the diagnosis of serious bacterial infections (SBIs) before and after implementing the new guideline.

**Results:**

We included 1,326 infants aged ≤59 days with FWS. Among infants aged ≤21 days, urine cultures increased from 49% to 67% (*p* = 0.001), blood cultures from 43% to 63% (*p* < 0.001), lumbar punctures from 16% to 33% (*p* = 0.003), and antibiotics from 38% to 57% (*p* = 0.002). Only 39 of 142 (28%) infants aged ≤21 days received recommended management. The SBI prevalence was 16.7% (95% CI, 11.0–23.8) and 17.6% (95% CI, 11.7–24.9) before and after the implementation, respectively. Among infants aged ≤59 days, there were 3 infants (0.6%; 95% CI, 0.1–1.7) in the pre-implementation period and 3 infants (0.6%; 95% CI, 0.1–1.7) in the post-implementation period with delayed treated urinary tract infections.

**Conclusions:**

Investigations and antibiotics increased significantly after implementation of the new guideline. However, doing more did not improve the diagnosis of SBIs. Thus, the low adherence to the new guideline may be considered justified. Future research should consider strategies to safely minimize interventions when managing infants with FWS.

## 1 Introduction

Fever is a common reason for evaluating infants in pediatric emergency departments (PEDs) (1). Many infants have no clinical symptoms or signs of infection, which is described as fever without a source (FWS). Serious bacterial infections (SBIs), often defined as urinary tract infection (UTI), bacteremia, and bacterial meningitis, are the causes of FWS in 7%–25% of infants aged ≤59 day (2–4). These infections are associated with high morbidity, thus, timely treatment is essential. Therefore, several guidelines for managing febrile infants aged ≤59 days have been developed over the last decades (5–8). Guidelines have been associated with better patient outcome and improved resource utilization (9–12). However, other studies have shown that guidelines did not improve the outcome of febrile infants but rather increased testing, antibiotic treatments, and hospitalizations (13–15). Additionally, new guidelines are often faced with skepticism from healthcare personnel, and their implementation can be challenging. Consequently, adherence to guidelines is often low and not sustained over time (16–18).

There are no national or regional guidelines for the management of infants with FWS in Sweden. A recently published study showed low rates of investigations and hospitalizations in infants aged ≤21 days and raised concerns about patient safety and missed SBIs (19). Thus, a new local hospital guideline for the management of term, previously healthy febrile infants aged ≤59 days was implemented in two PEDs in Sweden in 2018. This guideline was an adjustment of the “Step-by-Step” approach (5). To the best of our knowledge, only a few studies have investigated the impact of new guidelines on the management of febrile infants (12, 20). In this study, we aimed to describe the differences in the management and SBI diagnosis of febrile infants aged ≤59 days before and after the introduction of the new guideline.

## 2 Methods

### 2.1 Study design

A retrospective cohort study was conducted at two University PEDs in Sweden with approximately 35 000 annual visits together. Both PEDs are the only health facilities available for febrile infants aged ≤59 days in their catchment areas. The study period was from January 1, 2014, to December 31, 2021. The study was approved by the Regional Ethics Committee in Lund, Sweden (Dnr 2017/967).

### 2.2 Study population

All infants aged ≤59 days with “fever” registered as the main complaint in the electronic registration system were eligible for inclusion. Infants who were premature (<37 weeks at birth) and those who did not have a documented temperature of ≥38.0°C, either at PED or at home, were excluded. Furthermore, we excluded infants with comorbidities such as cardiovascular, neuromuscular, respiratory, and genitourinary tract disorders, and infants who were hospitalized or received antibiotics within the last 10 days. Infants with a clear focus of infection, such as in the upper respiratory tract, gastrointestinal tract, skin, or joints, were also excluded. Hence, only term, previously healthy infants with FWS, who should be managed according to the new guideline, were analyzed in this study. Revisits to PED within 10 days after the index visit if the infants were not admitted were also reviewed.

### 2.3 Study setting

None of the sites had specific clinical guidelines for infants with FWS before January of 2018 when the new clinical guideline was implemented. The management was individualized, mainly based on physician judgment. Routine practice included C-reactive protein (CRP), white blood cell (WBC) count, absolute neutrophil count (ANC), and urine dipstick. Febrile infants who were ill-appearing, with alarming symptoms, or altered test results were usually investigated further with blood and urine cultures, lumbar puncture (LP), were hospitalized, and received intravenous broad-spectrum antibiotics. Well-appearing infants, even those ≤28 days of age, with normal test results were often observed clinically for 12–24 h or discharged with instructions for a revisit the following day (18).

### 2.4 The new clinical guideline

**Figure 1:**
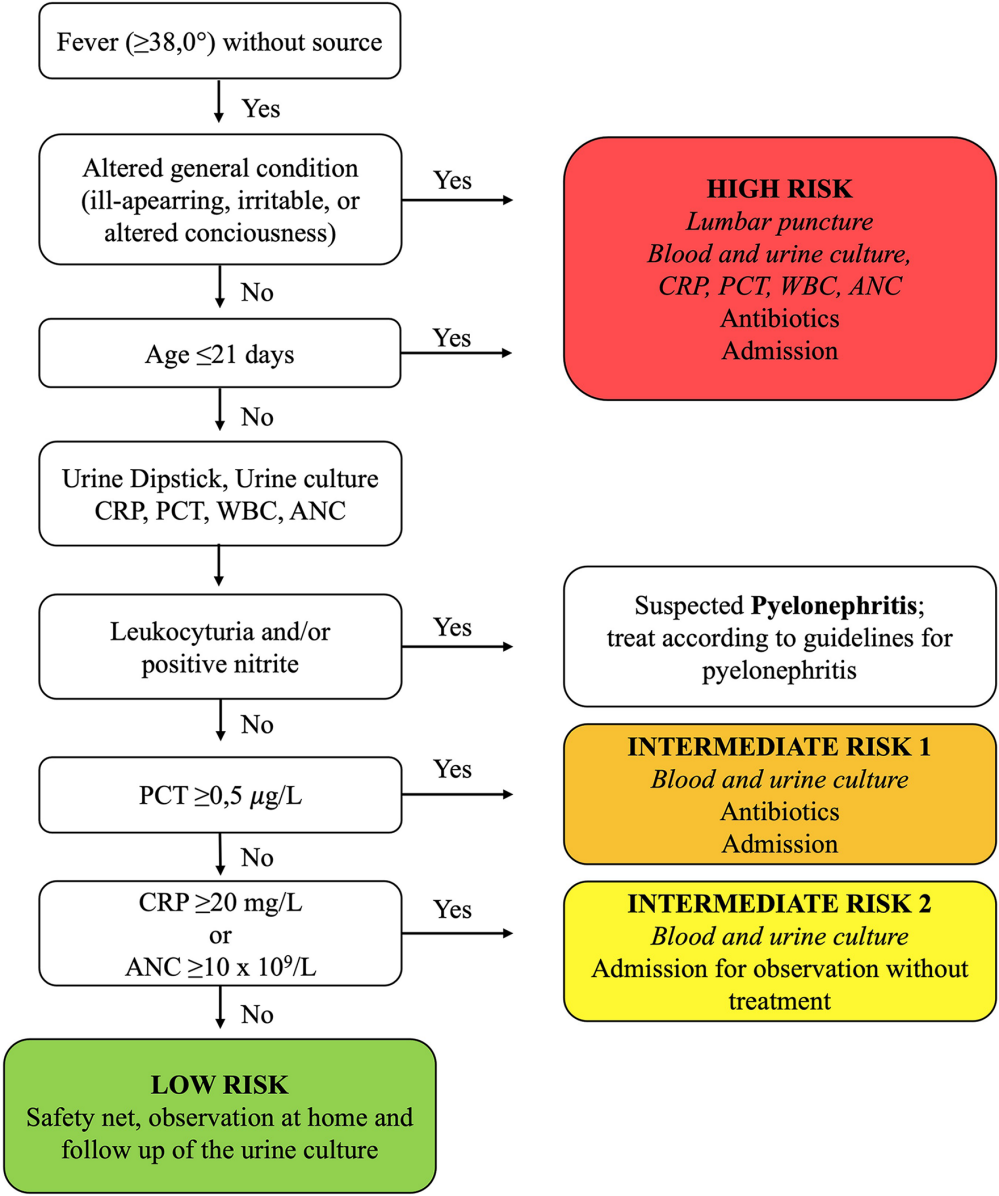
Summary of the new guideline for managing febrile infants aged ≤59 days with fever without source. Foot note: ANC, absolute neutrophile count; CRP, C-reactive protein; PCT, procalcitonin; WBC, white blood cell count.

The new clinical guideline was implemented in January 1, 2018 at both sites. The guideline stratified infants aged ≤59 days with FWS into four risk groups (high, low, intermediate 1, and intermediate 2) based on criteria such as age (≤21 days), clinical appearance, and laboratory results (Figure 1). Urine dipstick and culture, CRP, PCT, WBC and ANC count were recommended for all febrile infants aged ≤59 days. Additionally, the new guideline recommended LP, blood culture, and admission with parenteral antibiotics for all ill-appearing infants and those aged ≤21 days. The guideline was communicated through educational sessions for clinicians during meetings, and it was easily accessible at the hospital's internal guideline website and in a guideline's handbook.

### 2.5 Data collection

The Research Electronic Data Capture (REDCap) program hosted by Lund University (Lund, Sweden) was used to register the data. Data were collected retrospectively from the medical records. The following data were obtained: demographics (age, sex), temperature, duration of fever, symptoms, investigations [LP, blood, urine, and cerebrospinal fluid (CSF) cultures, urinalysis, CRP, WBC count, ANC], treatment with broad-spectrum antibiotics, admissions, revisits, delayed-treated SBIs, and mortality.

### 2.6 Outcome and study definitions

Primary outcome was SBI diagnosis, and secondary outcome was change in management and hospitalizations. Fever without a source was defined as a temperature ≥38.0°C, measured at the PED or at home, without any apparent site of infection (e.g., respiratory, gastrointestinal, skin, or joint). We registered infants as ill appearing if the following descriptions were used: septic, lethargic, irritable, ill appearing, somnolent, and non-responsive. Serious bacterial infections were defined as UTIs, bacteremia, or bacterial meningitis. Delayed-treated SBI was defined as any SBI in which broad-spectrum antibiotics were not administered during the initial approach. “Initial approach” was defined as any investigations or treatments performed or initiated at the PED. The definition of UTI was: (1) any amount of a single pathogen for suprapubic aspiration samples, (2) >100 000 colony-forming units per milliliter (cfu/ml) of a single pathogen for all urine sample methods, (3) 10 000–100 000 cfu/ml of a single pathogen combined with a urine dipstick positive for nitrite or leukocyte esterase in a sample obtained by catheterization or a “clean catch” method. This UTI definition was used as urine culture at the study sites are reported with three growth intervals: (1) <10 000 cfu/ml, (2) 10 000–100 000 cfu/ml, and (3) >100 000 cfu/ml. Bacterial meningitis was defined as a CSF culture or Polymerase Chain Reaction (PCR) test positive for bacterial pathogens. Bacteremia was defined as the growth of a bacterial pathogen in a blood culture. The presence of *Propionibacterium* spp., *Bacillus cereus* spp., diphteroids, micrococci, alpha-hemolytic streptococci, and coagulase-negative staphylococci were considered contaminants.

### 2.7 Data analysis

Data were analyzed using IBM SPSS Statistics version 28.0. Continuous normally distributed data were presented as mean with standard deviation, continuous non-normally data were presented as median with interquartile range (IQR), and categorical variables were reported as frequencies and percentages. The exact binominal interval method was used to calculate 95% confidence intervals (CIs). For comparison between groups in categorical variables, the Chi-Squared test was used. The level of significance was set at *p *< 0,05.

## 3 Results

We included 1,326 infants aged ≤59 days with FWS. Of these, 663 (50%) infants were evaluated before the implementation of the new clinical guideline and 663 (50%) were evaluated after. In the first period, 72 (11%, 95% CI 7–14) infants were classified as ill-appearing, compared to 39 (6%, 95% CI 4–8) in the second period (Table 1).

**Table 1:** Characteristics of infants ≤59 days with fever without a source before and after the implementation of a clinical guideline.

	Pre-implementation	Post-implementation
	*n* = 663	*n* = 663
Girls, *n* (%; 95% CI)	282 (43; 39–46)	293 (44; 40–48)
Age, median (IQR), days	39 (24–50)	38 (24–49)
Age ≤21 days, *n* (%; 95% CI)	144 (22; 19–25)	142 (21; 18–25)
Temperature home °C, mean (SD)	38.6 (0.5)	38.4 (0.4)
Temperature PED °C, mean (SD)	38.2 (2.2)	38.2 (0.7)
Afebrile at the PED, *n* (%; 95% CI)	191 (29; 25–32)	230 (35; 31–39)
Duration of fever, *n* (%; 95% CI)		
<6 h	505 (76; 73–79)	486 (73; 70–77)
6–12 h	85 (13; 10–16)	109 (16; 14–20)
12–24 h	45 (7; 5–9)	35 (5; 4–7)
24–48 h	19 (3; 2–4)	7 (1; 0–2)
>48 h	5 (1; 0–2)	14 (2; 1–4)
Unknown	3 (0; 0–1)	12 (2; 1–3)
Not well-appearing, *n* (%; 95% CI)	72 (11; 7–14)	39 (6; 4–8)

CI, confidence interval; IQR, interquartile range; SD, standard deviation; PED, pediatric emergency department.

Temperature home *n* = 586 and 597.

### 3.1 Difference in management of infants aged ≤21 days with FWS

After the implementation of the new clinical guideline, LP increased from 16% (95% CI, 10–23) to 33% (95% CI, 25–42), blood cultures from 43% (95% CI, 35–52) to 63% (95% CI, 55–71), and urine cultures from 49% (95% CI, 40–57) to 67% (95% CI, 59–75) in infants aged ≤21 days. Intravenous broad-spectrum antibiotic treatment increased from 38% (95% CI, 30–47) to 57% (95% CI, 48–65), whereas the number of hospitalizations did not change. Procalcitonin use increased from 9% (95% CI, 5–15) to 70% (95% CI, 62–77). The adherence rate to the guideline, including blood culture, urine culture and lumbar puncture, among infants aged ≤21 days was 28% (95% CI, 20–36) (Table 2).

**Table 2:** Management of infants ≤59 days with fever without a source before and after the implementation of a clinical guideline.

	0–21 days			22–59 days		
	Pre-implementation	Post-implementation	*p*	Pre-implementation	Post-implementation	*p*
	*n* = 144 *n* (%; 95%CI)	*n* = 142 *n* (%; 95%CI)		*n* = 519 *n* (%; 95%CI)	*n* = 521 *n* (%; 95%CI)	
Urine dipstick	112 (78; 70–84)	122 (86; 79–91)	0.070	441 (85; 82–88)	484 (93; 90–95)	<0.001
Positive^a^	30 (27; 19–36)	33 (27; 19–36)	0.883	102 (23; 19–27)	127 (26; 22–30)	0.307
ANC	72 (50; 42–58)	99 (70; 62–77)	<0.001	238 (46; 42–50)	291 (56; 52–60)	0.001
CRP	123 (85; 79–91)	127 (89; 83–94)	0.295	458 (88; 85–91)	420 (81; 77–84)	<0.001
PCT	13 (9; 5–15)	99 (70; 62–77)	<0.001	39 (8; 5–10)	323 (62; 58–66)	<0.001
Urine culture	70 (49; 40–57)	95 (67; 59–75)	0.001	198 (38; 34–43)	339 (65; 61–69)	<0.001
Blood culture	62 (43; 35–52)	89 (63; 55–71)	<0.001	140 (27; 23–31)	145 (28; 24–32)	0.830
Lumbar puncture	23 (16; 10–23)	47 (33; 25–42)	0.003	27 (5; 4–8)	26 (5; 3–7)	0.848
All three^b^	19 (13; 8–20)	39 (28; 20–36)	0.003	19 (4; 2–6)	19 (4; 2–6)	0.904
Antibiotics^c^	55 (38; 30–47)	80 (57; 48–65)	0.002	102 (20; 16–23)	114 (22; 18–26)	0.420
Hospitalized	95 (66; 58–74)	100 (70; 62–78)	0.397	243 (47; 43–51)	181 (35; 30–39)	<0.001
Revisits	21 (43; 29–58)	11 (26; 14–42)	0.097	113 (41; 35–47)	99 (29; 24–34)	0.002

ANC, absolute neutrophil count; Contaminated blood culture, Coagulase-negative staphylococci, *Propionibacterium* spp, *Bacillus cereus* spp, micrococci, alpha hemolytic streptococci, diphtheroids; CRP, C-reactive protein; PCT, procalcitonin; Revisits, percentage of infants not hospitalized; WBC, white blood cell count.

^a^ Positive for nitrite and/or leukocyte esterase, expressed as percentage of all urine dipsticks obtained.

^b^ Urine culture, blood culture and lumbar puncture.

^c^ Intravenous antibiotics i.e., cefotaxime, ampicillin, gentamicin.

### 3.2 Differences in management of infants aged 22–59 days with FWS

In infants aged 22–59 days, urine cultures increased from 38% (95% CI, 34–43) to 65% (95% CI, 61–69), whereas the number of lumbar punctures and blood cultures did not change. The number of hospitalizations decreased from 47% (95% CI, 43–51) to 35% (95% CI, 30–39). The use of PCT increased from 8% (95% CI, 5–10) to 62% (95% CI, 58–66) (Table 2).

### 3.3 Differences in management according to clinical appearance and fever

Differences in management according to clinical appearance and the presence of fever were analyzed across the entire cohort, encompassing both pre- and post-implementation periods of the new clinical guideline. Among infants aged ≤21 days, LP was performed 7 times more often in febrile, ill-appearing infants and 3 times more often in febrile, well-appearing infants than in afebrile, well-appearing infants (Table 3). The rates of antibiotics and hospitalizations were also higher in ill-appearing or febrile infants than in well-appearing or afebrile infants.

**Table 3:** Lumbar punctures, antibiotics, and hospitalizations at the initial approach^a^ of infants aged ≤59 days with fever without a source according to the general appearance and presence of fever at presentation.

	0–21 days *n* = 286		22–59 days *n* = 1,040	
	Well-appearing *n* = 248 *n* (%; 95% CI)	Ill-appearing^e^ *n* = 38 *n* (%; 95% CI)	Well-appearing *n* = 967 *n* (%; 95% CI)	Ill-appearing^e^ *n* = 73 *n* (%; 95% CI)
Febrile^b^ PED^c^	156	34	651	64
Lumbar Puncture	40 (26; 19–33)	22 (65; 47–80)	22 (3; 2–5)	23 (28; 18–39)
Antibiotics^d^	81 (52; 44–61)	28 (82; 66–93)	144 (22; 19–26)	44 (69; 56–80)
Hospitalizations	123 (79; 72–85)	33 (97; 85–100)	314 (48; 44–52)	54 (84; 73–92)
Afebrile^b^ PED^c^	92	4	316	9
Lumbar Puncture	8 (9; 4–16)	0 (0; 0–60)	2 (1; 0–2)	4 (44; 14–79)
Antibiotics^d^	20 (22; 14–32)	4 (100; 40–100)	21 (7; 4–10)	7 (78; 40–97)
Hospitalizations	35 (38; 28–49)	4 (100; 40–100)	47 (15; 11–19)	9 (100; 66–100)

^a^ Initial approach, investigations, and antibiotic treatment performed at the PED or the ward planned by the PED physician.

^b^ Febrile, Temperature ≥38.0°C; Afebrile, Temperature <38.0°C.

^c^ PED, Pediatric Emergency Department.

^d^ Antibiotics, intravenous antibiotics i.e., cefotaxime, ampicillin, gentamicin.

^e^ Ill-appearing, documented in the medical record as any of: ill-appearing, irritable, somnolent, lethargic, non-responsive, or septic.

### 3.4 Differences in SBI diagnosis

The prevalence of SBIs among infants aged ≤21 days was 16.7% (95% CI, 11.0–23.8) and 17.6% (95% CI, 11.7–24.9) in the pre- and post-implementation periods, respectively (Table 4). There were no infants aged ≤21 days with delayed treated SBIs in either the pre- or postimplementation period. One infant aged ≤21 days died during the pre-implementation period because of disseminated herpes virus infection. In the 22–59 days group, the rate of SBI was 9.4% (95% CI, 7.1–12.3) and 11.7% (95% CI, 9.1–14.8) in the pre- and post-implementation periods, respectively. Three infants (0.6%; 95% CI, 0.1–1.7) in the pre-implementation period and three (0.6%; 95% CI, 0.1–1.7) in the post-implementation period with UTI were not identified and treated at their index visit.

**Table 4:** Diagnosis of SBIs in infants ≤59 days with fever without a source before and after the implementation of a clinical guideline.

	0–21 days		22–59 days	
	Pre-implementation	Post-implementation	Pre-implementation	Post-implementation
	*n* = 144	*n* = 142	*n* = 519	*n* = 521
SBI Total	24 (16.7; 11.0–23.8)	25 (17.6; 11.7–24.9)	49 (9.4; 7.1–12.3)	61 (11.7; 9.1–14.8)
UTI^a^	22 (15.3; 9.8–22.2)	22 (15.5 10.0–22.5)	47 (9.1; 6.7–11.9)	60 (11.5; 8.9–14.6)
Bacteremia^a^	3 (2.1; 0.4–6.0)	5 (3.5; 1.2–8.0)	3 (0.6; 0.1–1.7)	2 (0.4; 0.0–1.4)
Meningitis^a^	1 (0.7; 0.0–3.8)	2 (1.4; 0.2–5.0)	0 (0.0; 0.0–0.7)	0 (0.0; 0.0–0.7)

PED, Pediatric Emergency Department; SBI, Serious Bacterial Infection; UTI, Urinary Tract Infection; Meningitis, Bacterial Meningitis.

^a^ All cases (isolated or in any combination), because of the combinations the sum of UTI, Meningitis and Bacteremia is higher than the number of SBI Total.

## 4 Discussion

We investigated the impact of implementing a new clinical guideline on the management of infants aged ≤59 days with FWS and the diagnosis of SBIs. Adherence was low, with only a small proportion of febrile infants receiving the recommended management. The new guideline increased the number of investigations and antibiotic treatments, but there was no difference in missed SBI diagnosis before or after implementation.

After the implementation of the new management guideline, LPs increased by 100%, blood cultures by 50%, urine cultures by 37%, and antibiotics by 50% in infants aged ≤21 days. This aligns with findings from other studies that reported an increase in the number of investigations following the introduction of guidelines for febrile infants (10–12). However, despite this increase, only 28% of febrile infants ≤21 days in our study were managed according to the new guideline, primarily due to low adherence to perform lumbar puncture. Gomez et al. evaluated the implementation of the “Step by Step” approach and found an adherence rate of 63% in infants aged <15 days with FWS (12). Similarly, various studies have reported adherence rates to local guidelines between 45%–66% (15, 20, 21). Low compliance with recommendations was also found in 2 surveys conducted in Canada and the USA (22, 23). Barriers to implementing new guidelines have been investigated in several studies. Fischer et al. reported that factors related to physicians' knowledge and attitudes (encompassing skills, learning culture, guideline awareness, motivation), to the guideline (accessibility, applicability, and complexity), and organizational considerations (lack of resources or collaboration) are the most common hindrances to following guidelines (17).

However, there is limited knowledge of the factors that influence adherence to guidelines for febrile infants. We found that the rates of LPs, antibiotics, and admissions were lower in well-appearing and/or afebrile infants compared to those who were ill-appearing and/or still febrile, consistent with findings from previous research (24–26). Thus, it was hypothesized that the clinical appearance and the presence of fever during examination might be factors that influence compliance with guidelines (21, 27). This hypothesis is supported by a recent qualitative study, which identified that physicians relied on their clinical judgement to decide whether to perform a LP and admit the infants for parenteral antibiotics (18). Furthermore, the possibility of adequate follow-up has also been suggested as a contributing factor to low adherence to guidelines (15). Our study observed a high rate of revisits (29%–41%) during the pre- and post-implementation periods. Sweden has free universal healthcare for children. Additionally, access to the study PEDs is easy, with the majority of the catchment population living within 30 min. We believe that the possibility of revisits combined with easy access could be a patient-safe alternative to the recommended routine extensive investigations and hospitalizations. Guidelines derived in Spain or the USA or an “one size fits all” approach may not be optimal for patients in all settings, especially in settings with different characteristics. Furthermore, what considers as “medical consensus” can vary between countries, medical groups, and time periods (28).

Additionally, our study identified only one case of meningitis and three cases of missed UTIs in the four years prior to the implementation of the guideline. Therefore, physicians may not have been exposed to unfavorable outcomes or cases of missed meningitis, and consequently might not have seen any need to change an approach that had worked well. Aronson et al. reported that physicians' risk aversion and previous experience with unfavorable outcomes resulted in increased compliance with guidelines for febrile infants (29). Furthermore, we identified a 63% increase in urine cultures. This was the result of the new guideline, which recommended urine culture for all febrile infants aged ≤59 days irrespective of the urine dipstick result, as recommended by all international guidelines in 2018. However, the latest guideline by the American Academy of Pediatrics (AAP) released in 2021 recommends urine culture only with positive urinalysis (8). Thus, physicians' praxis, to not routinely perform urine culture, prior to the new guideline, was aligned with scientific evidence. Studies have shown that guidelines can quickly be outdated and are quite slow to incorporate new knowledge or introduce new tests (30). Also, their quality is often unsatisfactory, as a study by Grilli et al. showed that only 5% of the 431 guidelines reviewed met all necessary quality criteria (31, 32).

Another interesting finding of this study is that the prevalence and number of delayed treated SBIs did not change after the implementation of the new guideline, despite the 50%–100% increase in investigations and antibiotic treatments. Previous studies did not find any association between improved outcomes or lower rates of missed meningitis/bacteremia with extensive investigations, antibiotic treatments, or hospitalizations (20, 21, 27, 33). Pantell et al. concluded that “‘practitioners relying on their clinical judgment were at least as sensitive in treating bacteremia and bacterial meningitis as the current guidelines”' (15). Medical judgment and patient care are complex processes that cannot be reduced to algorithms with sequencies of binary (yes/no) alternatives.^33^ Such approaches not only overlook patients' individuals needs or characteristics, but often result in encouraging ineffective and wasteful interventions (34). Investigations, antibiotics, and admissions are associated with iatrogenic complications, adverse events, hospital acquired infections, increased costs for the healthcare system, and financial burden and stress for the families (13, 35–38). Hence, guidelines by increasing investigations, antibiotics, and admissions can result in “patient harm.” Such concerns that guidelines might result in more harm than benefit for the patient and that the best way to prevent patient harm might be by doing less, have already been raised in the literature (39–41). If physicians had followed the guideline in the study PEDs, febrile infants would have been exposed to 2- to 4-fold more LPs, antibiotic treatments, and hospitalizations without identifying any case of meningitis or bacteremia. Thus, physician's noncompliance with the guideline seems justifiable.

Our study has some limitations. First, the inclusion method may have caused sampling bias since there is a risk that we did not include all infants aged ≤59 days with fever. It is possible that another chief complaint (i.e., vomiting, refusal to feed, fatigue) was registered in the PED's electronic system, even though the infant was febrile. Second, the retrospective nature of the study may have compromised the quality of data regarding the source and duration of fever, as well as the general appearance. Third, urine, blood, and CSF cultures were not collected from all infants, thus, SBIs may have been missed. However, we believe that the risk of missed SBIs in our study is low given a follow-up period of 10 days and that the electronic journal system is common in all regional hospitals, and primary or private health facilities do not see febrile infants aged ≤59 days. Fourth, our study was conducted in two PEDs, thus, the results may not be generalizable to different settings. Additionally, our data do not allow conclusions to be drawn regarding the factors that influence physicians’ compliance with the guidelines. Finally, due to the retrospective nature and short duration of the study, we were unable to collect data regarding possible adverse events due to investigations, antibiotics, or hospitalizations. Larger and longer studies are needed to investigate possible patient harm due to guidelines.

## 5 Conclusion

Physicians did not fully comply with the new guideline, especially in routinely performing LP, administering antibiotics, and hospitalizing febrile infants aged ≤21 days. Despite low adherence, the new guideline significantly increased investigations and antibiotics. However, doing more did not lead to better diagnosis of SBIs. Hence, doing more may be harmful for infants and costly for families and the health care system. Thus, physicians' noncompliance with the guideline was likely justified. We believe that current research and dialogue regarding the management of febrile infants should consider more on how we can safely do less.

## Data Availability

The raw data supporting the conclusions of this article will be made available by the authors, without undue reservation.
